# Supplementary figures and images for: A novel silver-ruthenium-based antimicrobial kills Gram-negative bacteria through oxidative stress-induced macromolecular damage
Source: mSphere. 2025 May 30;10(6):e00017-25. doi: 10.1128/msphere.00017-25 (PMC12188735; doi:10.1128/msphere.00017-25)

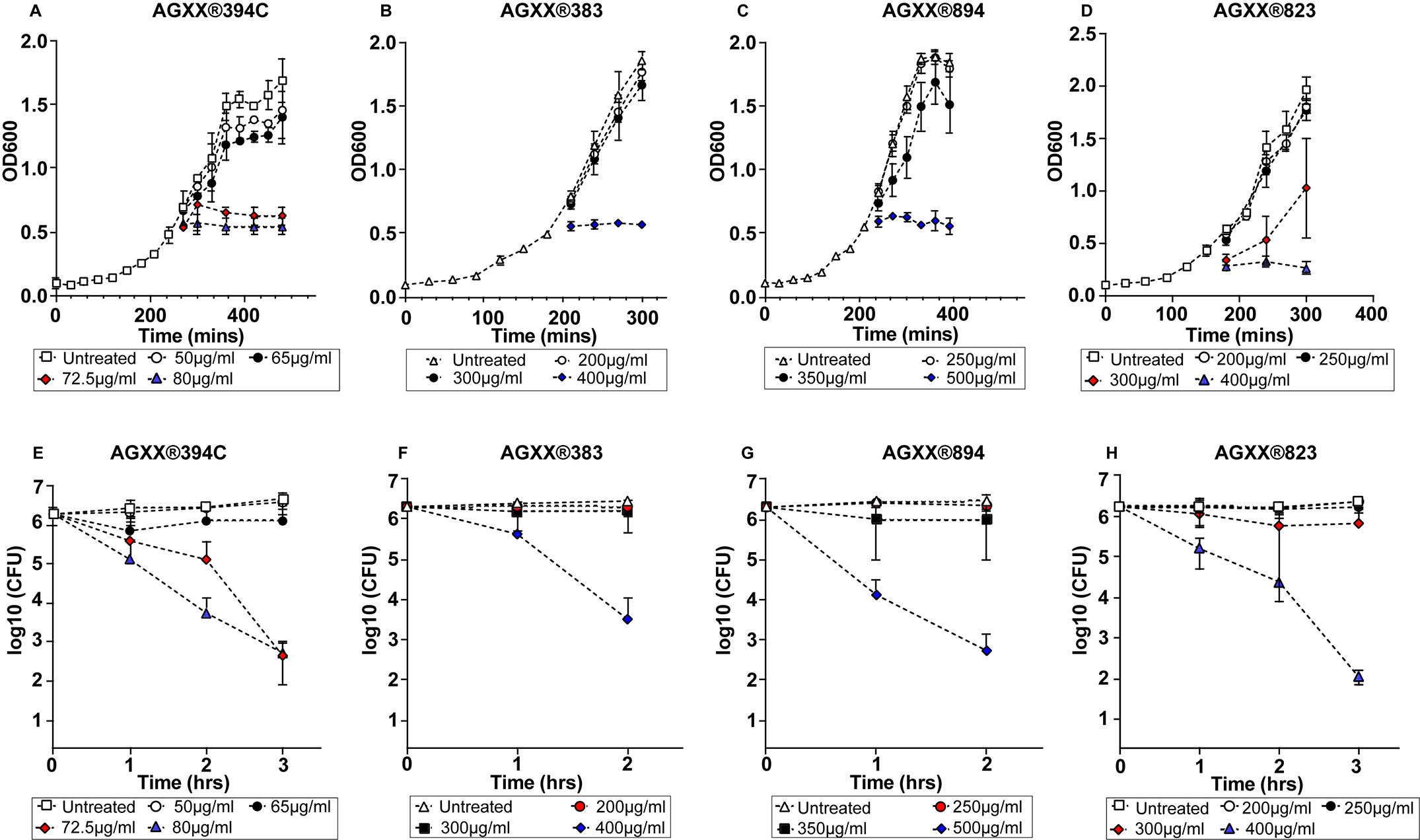

Supplement: Figure S1 — AGXX formulations differ in their antimicrobial activities. [file msphere.00017-25-s0001.tif]

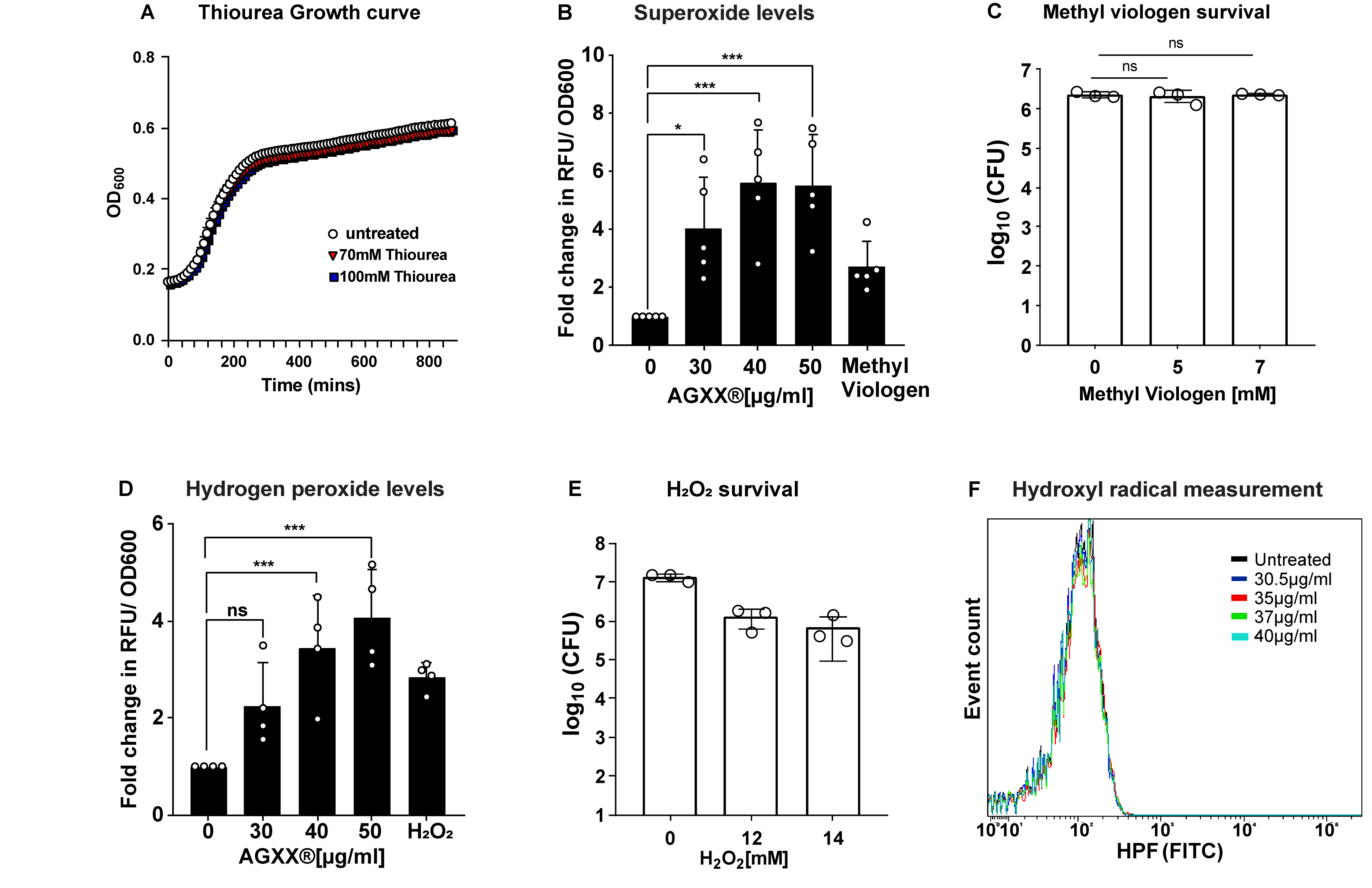

Supplement: Figure S2 — ROS measurements. [file msphere.00017-25-s0002.tif]

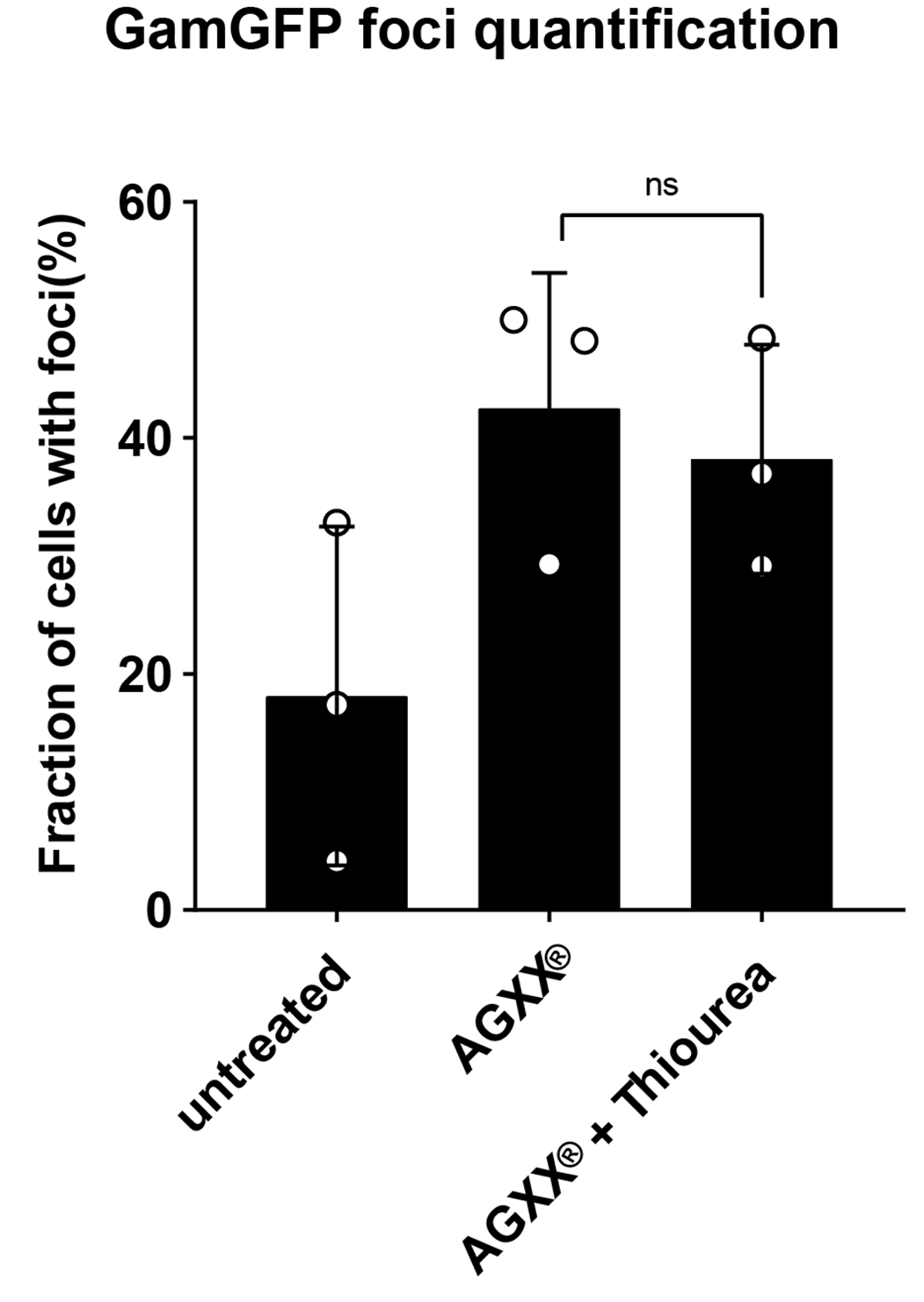

Supplement: Figure S3 — Gam-sfGFP foci with thiourea. [file msphere.00017-25-s0003.tif]

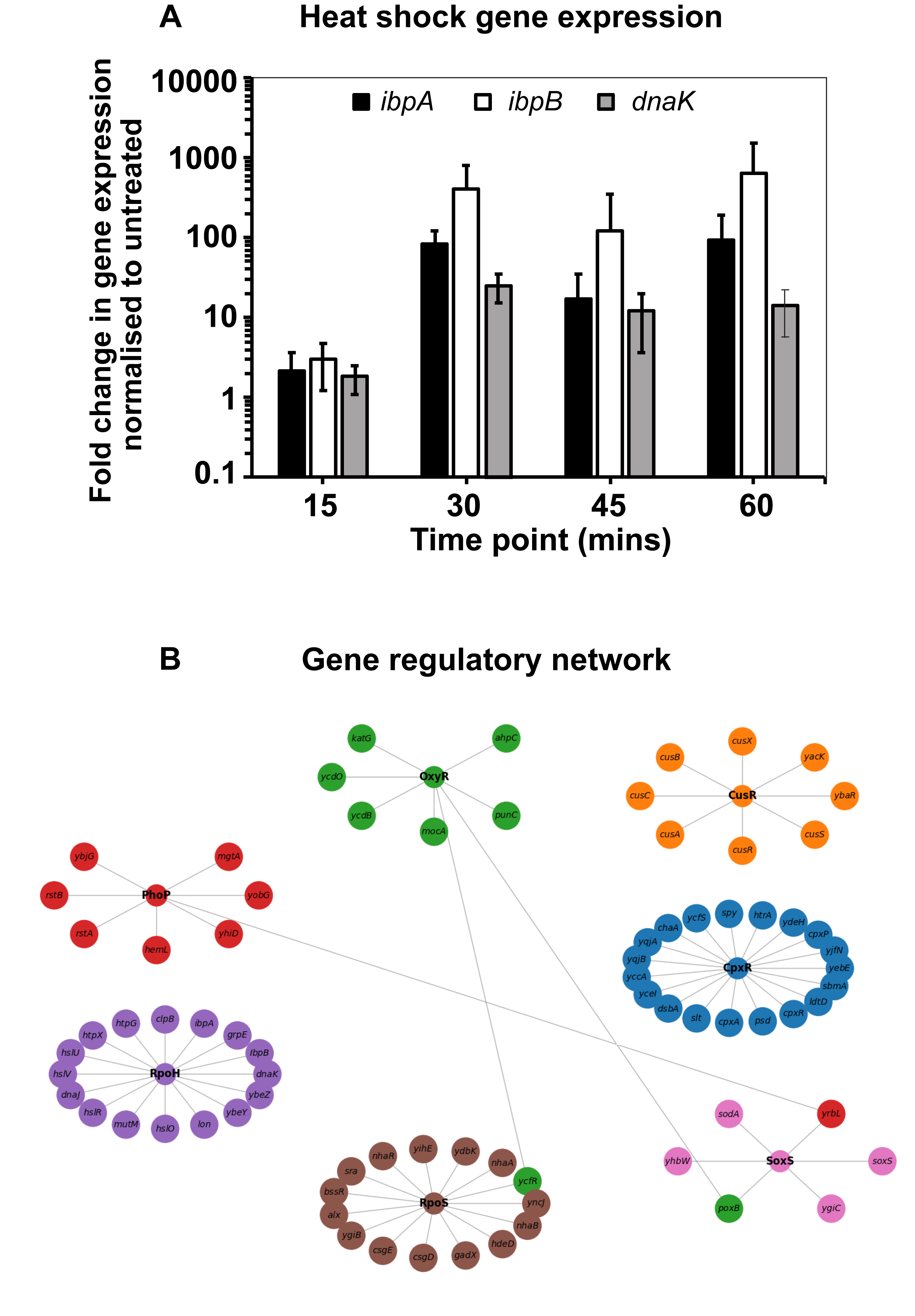

Supplement: Figure S4 — Transcriptomics. [file msphere.00017-25-s0004.tif]

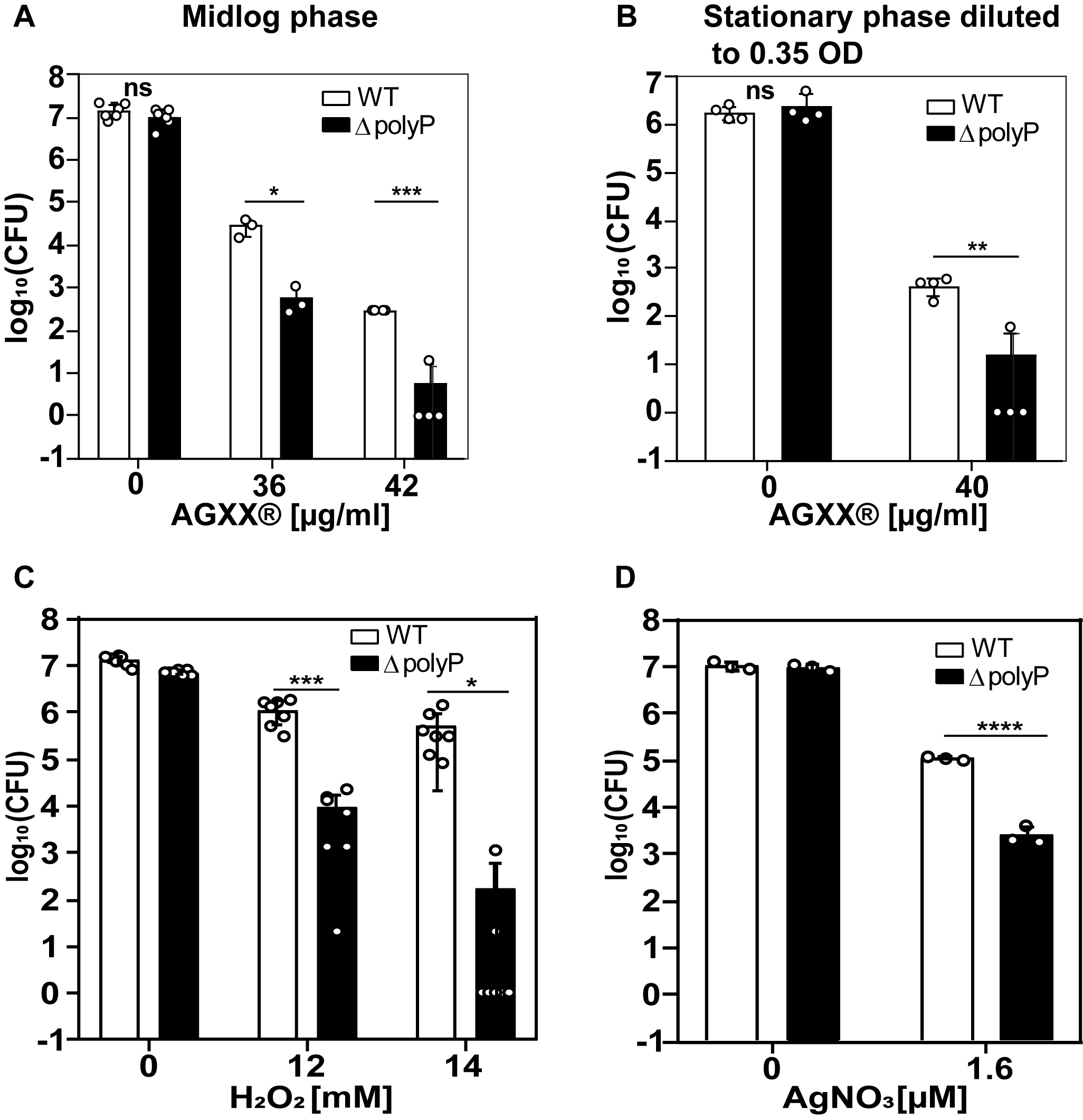

Supplement: Figure S5 — PolyP. [file msphere.00017-25-s0005.tif]
