# Supplementary material for: A novel silver-ruthenium-based antimicrobial kills Gram-negative bacteria through oxidative stress-induced macromolecular damage
Source: mSphere. 2025 May 30;10(6):e00017-25. doi: 10.1128/msphere.00017-25 (PMC12188735; doi:10.1128/msphere.00017-25)
Supplement: Legends — for Figures S1-S5 and Tables S1-S4. [file msphere.00017-25-s0006.pdf]

SUPPLEMENTARY INFORMATION

Supplementary Fig. S1: AGXX® formulations differ in their antimicrobial activities. Growth and survival studies were performed in UPEC strain CFT073, which was grown in MOPSg media to mid-log phase and treated with the indicated concentrations of AGXX®394C (**A; E**), AGXX®383 (**B; F**), AGXX®894 (**C; G**), and AGXX®823 (**D; H**), respectively. (**A-D**) Absorbance at 600 nm (OD_{600}) was recorded every 30 mins for 4 hrs (n=3-4, ±S.D.). (**E-H**) For assessment of bacterial killing, samples were taken every 60 minutes and serially diluted in PBS. Five μl of serial dilutions were spotted onto LB agar for colony forming units (CFU) counts after overnight incubation (n=4-7, ±SD)

Supplementary Fig. S2: (**A**) Overnight cultures of CFT073 were diluted ~25-fold into fresh MOPSg, grown at 37 ˚C until late logarithmic phase (OD600= ~2), diluted to an OD_{600}= 0.02, and cultivated in the presence of the indicated thiourea concentrations. Growth was monitored at 600 nm for 16 hrs in the Tecan plate reader. (**B; D**) Intracellular superoxide (**B**) and hydrogen peroxide (**D**) levels were quantified by DHE and Amplex Red, respectively (n=4-5, ±S.D.). **(C; E)** Bacterial survival after methyl viologen (**C**) and hydrogen peroxide (**E**) stress was determined by serial dilution in PBS, spot-titered on LB agar, and incubated for 20 hrs for CFU counts; (n=3, ±S.D., one-way ANOVA, Sidak's multiple comparison test; ns = P > 0.05, * P < 0.05, ** P < 0.01, *** P < 0.001). (**F**) Intracellular hydroxyl radical levels were quantified in exponentially growing CFT073 cultures exposed to the indicated concentration of AGXX®394C for 60 min by staining cells with 10 μM HPF. Dye fluorescence was measured via flow cytometry after 30 min of incubation. One representative of 3 independent experiments of similar outcomes.

Supplementary Fig. S3: Pretreatment with thiourea does not rescue AGXX®-induced DNA damage. Exponentially growing cells expressing Gam-sfGFP were split into two part, of which one was pretreated with 70 mM thiourea. Cells were either left (i) untreated or (ii) treated with 40 μg/ml AGXX®394C for 180min. Samples were washed, incubated with DAPI in the dark for 15 min, and visualized by confocal microscopy. Gam-sfGFP foci were quantified by counting the number of foci per cell (n=3, ±S.D., one-way ANOVA, Sidak's multiple comparison test; ns = $P >$ 0.05).

Supplementary Fig. S4: Time-course of the transcriptional response of UPEC to AGXX® treatment and classification of differentially expressed genes during AGXX® stress into regulons. (A) Exponentially growing CFT073 cultures were exposed to 250 μg/ml AGXX®823 and samples collected at the indicated timepoints for RNA extraction, removal of genomic DNA, and reverse transcription of mRNA into cDNA. qRT-PCR analysis was performed for select heat shock genes and normalized to the housekeeping gene *rrsD* and untreated samples (n= 4, ±S.D.). **(B)** Differentially expressed genes during AGXX® stress are classified into regulons based on the RegPrecise database (https://regulondb.ccg.unam.mx/). Center nodes represent transcriptional regulator or sigma factor while downstream target genes are represented by the surrounding nodes in the same color. The strong oxidative stress, metal stress and heat shock responses induced by AGXX® are mediated by SoxS, OxyR, CusR, and RpoH regulons. The induction of the RpoH, CpxR and PhoP regulons further emphasize general stress and cell envelope stress in AGXX®-treated CFT073.

Supplementary Fig. S5: Polyphosphate protects UPEC from ROS and silver. Exponentially growing CFT073 and ΔpolyP cells were exposed to the indicated concentrations of AGXX® for (**A**) 180 mins , (**B**) stationary phase cultures diluted to 0.35 OD for 150 mins, (**C**) the indicated concentrations of hydrogen peroxide (**D**) and silver nitrate for 180 min before samples were

serially diluted in PBS, spot-titered on LB agar, and incubated for 20 hrs for CFU counts (n= 3-7, ±S.D.). student t-test; ns = $P > 0.05$, * $P < 0.05$, *** $P < 0.001$, **** $P < 0.0001$.)

Supplementary Table S1: Quantification of live-dead cells, IbpA-GFP foci, and Gam-GFP foci. Cells were cultivated and treated as described in the materials & methods. Quantification was performed blindly.

Supplementary Table S2: AGXX® exposure of UPEC elicits significant changes in global gene expression. (A) Exponentially growing CFT073 cells were incubated with a sublethal concentration of AGXX®394C for 30 min. Transcription was stopped by the addition of ice-cold methanol. Reads were aligned to the CFT073 reference genome (accession number: AE014075). Data are visualized as a ratio/intensity scatter plot (M/A-plot) of differentially expressed genes in AGXX®-treated CFT073 cells.

Supplementary Table S3: UPEC-specific genes differentially expressed in AGXX®-treated UPEC. Exponentially growing CFT073 cells were incubated with a sublethal concentration of AGXX®394C for 30 min. Transcription was stopped by the addition of ice-cold methanol. Reads were aligned to the CFT073 reference genome (accession number: AE014075). Differentially expressed UPEC-genes are listed.

Supplementary Table S4: Strains and oligos used in this study.
