# Supplementary material for: A novel silver-ruthenium-based antimicrobial kills Gram-negative bacteria through oxidative stress-induced macromolecular damage
Source: mSphere. 2025 May 30;10(6):e00017-25. doi: 10.1128/msphere.00017-25 (PMC12188735; doi:10.1128/msphere.00017-25)
Supplement: Table S3 — RNAseq UPEC specific genes. [file msphere.00017-25-s0009.docx]

|  | UPREGULATED UPEC-SPECIFIC GENES | | |
| --- | --- | --- | --- |
| Gene Name | **Gene ID** | **Polypeptide** | **Log_2_ (FC)** |
| eschColi_CFT073_refSeq_c1936 | *c1936* | Type-1 fimbrial protein, A chain precursor | 5.56705157 |
| eschColi_CFT073_refSeq_c1935 | *c1935* | Chaperone protein fimC precursor | 5.206073902 |
| eschColi_CFT073_refSeq_c1934 | *c1934* | Outer membrane usher protein fimD precursor | 5.021445923 |
| eschColi_CFT073_refSeq_c1429 | *ycgW* | anti-adapter protein IraM | 4.4251792 |
| eschColi_CFT073_refSeq_c1507 | *c1507* | Hypothetical protein | 3.731820408 |
| eschColi_CFT073_refSeq_c3432 | *c3432* | Transposase | 2.953526876 |
| eschColi_CFT073_refSeq_c3433 | *c3433* | Hypothetical protein | 2.941273877 |
| eschColi_CFT073_refSeq_c1432 | *c1432* | Hypothetical protein | 2.448825284 |
| eschColi_CFT073_refSeq_c0086 | *c0086* | Transposase | 2.074589766 |
| eschColi_CFT073_refSeq_c0198 | *c0198* | Transposase | 2.074589766 |
| eschColi_CFT073_refSeq_c1291 | *c1291* | Hypothetical protein | 2.074589766 |
| eschColi_CFT073_refSeq_c1384 | *c1384* | Hypothetical protein | 2.074589766 |
| eschColi_CFT073_refSeq_c2622 | *c2622* | Transposase | 2.074589766 |
| eschColi_CFT073_refSeq_c3152 | *c3152* | Hypothetical protein | 2.074589766 |
| eschColi_CFT073_refSeq_c3031 | *c3031* | SinH homolog | 2.0670212 |
| eschColi_CFT073_refSeq_c4760 | *c4760* | Hypothetical protein | 1.569762462 |
| eschColi_CFT073_refSeq_c3230 | *proV* | glycine betaine ABC transporter ATP binding subunit ProV | 1.569259081 |
| eschColi_CFT073_refSeq_c1721 | *yciC* | Hypothetical protein yciC | 1.558817956 |

| DOWNREGULATED UPEC-SPECIFIC GENES | | | |  |
| --- | --- | --- | --- | --- |
| Gene Name | **Gene ID** | **Polypeptide** | **Log_2_ (FC)** | |
| eschColi_CFT073_refSeq_c3566 | *c3566* | Hypothetical protein | **-3.654584304** | |
| eschColi_CFT073_refSeq_c3698 | *kpsM* | KpsM protein | **-3.484472599** | |
| eschColi_CFT073_refSeq_c3695 | *c3695* | Hypothetical protein | **-3.45453464** | |
| eschColi_CFT073_refSeq_c3697 | *kpsT* | KpsT protein | **-3.154805693** | |
| eschColi_CFT073_refSeq_c3694 | *c3694* | Hypothetical protein | **-2.444767322** | |
| eschColi_CFT073_refSeq_c4581 | *c4581* | Hypothetical protein | **-1.984282372** | |
| eschColi_CFT073_refSeq_c4739 | *c4739* | Conserved hypothetical protein | **-1.692534671** | |
| eschColi_CFT073_refSeq_c4502 | *c4502* | Putative antiporter | **-1.603557113** | |
| eschColi_CFT073_refSeq_c1267 | *c1267* | Hypothetical protein | **-1.559625977** | |
